# The Effect of the Extrusion Ratio on Load and Die Wear in the Extrusion Process

**DOI:** 10.3390/ma16010084

**Published:** 2022-12-22

**Authors:** Stanisław Kut, Irena Nowotyńska

**Affiliations:** 1 Department of Materials Forming and Processing, Rzeszow University of Technology, Al. Powst. Warszawy 8, 35-959 Rzeszów, Poland; 2 Department of Computer Engineering in Management, Rzeszow University of Technology, Al. Powst. Warszawy 8, 35-959 Rzeszów, Poland

**Keywords:** extrusion, extrusion ratio, die load, die wear

## Abstract

In this paper, regarding the example of concurrent extrusion of a round bar, the results of a comparative analysis of the impact of the extrusion ratio R on process parameters such as die wear intensity, extrusion force, stress intensity in the die, and its elastic deformation during extrusion are presented. The tests were carried out in a wide range of the R (1.5–11), assuming a constant outer diameter of the die. A comparative analysis of the wear was carried out in the area of the die corner radius and the calibration strip since the wear is the highest there. Based on the result obtained, it was shown that the increase in the extrusion force was not proportional to the increase in the extrusion ratio, and this could be described by a logarithmic equation. On the other hand, the value of equivalent stresses in the area of the die corner depends, to a relatively small extent, on the value of the extrusion ratio. However, due to the deformation of the die, it is most advantageous to use an extrusion ratio greater than or equal to three. Based on the wear analysis, it was estimated that the increase in the extrusion ratio R from 3 to 7 caused a more than 2.5 times increase in tool wear. In turn, the implementation of the extrusion process with the use of R = 11 caused a 4.5-fold increase in the average depth of die wear compared to its wear using the extrusion ratio R = 3.

## 1. Introduction

Extrusion is the basic method of producing pipes, rods, and profiles from metals and their alloys. Extrusion is a plastic forming process in which very high degrees of deformation can be achieved without compromising the consistency of the material. The main limitation of the amount of deformation that can be obtained in one pressing operation is the strength of the tools [1]. Punches and extrusion dies are among the most loaded tools among those used in forming processes [2,3]. This results in high requirements for tool materials, their heat treatment, as well as construction and accuracy of workmanship.

The extrusion ratio R is the basic parameter characterizing the extrusion process:

(1) $$R=\frac{A_{1}}{A_{2}}=\frac{D_{1}^{2}}{D_{2}^{2}}\;,$$

where: A_1_—cross-sectional area of the billet, A_2_—cross-sectional area of the product, D_1_—diameter of the billet, and D_2_—diameter after extrusion.

The value of the extrusion ratio R affects not only the degree of deformation and the flow character of the extruded material, but also the strength parameters of this process. With an increase in the extrusion ratio R value, the force needed for extrusion grows, and thus the pressure of the formed material on the tools [4,5]. Consequently, the growth in force and flow velocity not only increases the load on the die, but also causes a significant increase in the intensity of its wear. At the same time, the extrusion ratio R influences the material properties of the extruded elements, including operational properties, strength, and microstructure [6,7]. In work [8], the effects of die land lengths on the die-shaped profiles and on the extrusion pressures were investigated and presented. The extrusion pressure contributions due to the die land evaluated theoretically for the shaped sections considered are found to increase with die land lengths for any given percentage reduction, and they also grow with increasing percentage of die reductions at any given die land length. Therefore, the influence of the extrusion ratio R should be considered in two aspects. The first one concerns the influence of the extrusion ratio on the operational properties, strength, and microstructure of extruded elements. The second one concerns the impact of the extrusion ratio R on the strength and wear of extrusion tools. The extrusion factor plays an important role in the production of extruded parts [9]. For example, in the case of the extrusion of magnesium alloys, the extrusion parameters, especially the extrusion ratio R, can significantly affect the microstructure, and thus determine the mechanical properties of the manufactured materials [10,11]. The study [12] investigates the effects of extrusion ratio and temperature on the microstructure and mechanical properties of as-extruded Mg-11.5Gd-4.5Y-(1Nd/1.5Zn)-0.3Zr alloys. In turn, in work [13], the influence of the extrusion temperature on the microstructure and mechanical behavior of the duplex Mg–Li–Al–Sr alloy was investigated. The extrusion ratio has a much greater impact on the fragmentation of the microstructure of magnesium alloys than the extrusion speed and temperature [14]. Along with the increase in the extrusion ratio R, the microstructure fragmentation [15] increases and, consequently, the strength parameters of alloys such as yield strength (YS) and ultimate tensile strength (UTS) grow. In works [16,17], the effect of extrusion ratio on the microstructure and mechanical properties of the as-extruded Mg–6Sn–2Zn–1Ca (TZX621) alloy and of the Mg–8Li–3Al–2Zn–0.5Y alloy with a duplex structure was investigated. The effect of extrusion ratio on the microstructure and mechanical properties of the TZX621 alloy was investigated [18]. When the extrusion ratio was increased from 6 to 16, the mechanical properties of the as-extruded TZX621 alloy were improved comprehensively. Tong et al. [19] reported that the effect of extrusion ratio on the mechanical properties of Mg–Zn–Ca alloys was not obvious, which was derived from the competitive mechanism between the fine grain strengthening and texture weakening effect. In work [20], the effect of extrusion ratio on the microstructure and tensile properties of the Mg–Sm–Zn–Zr alloy was investigated in detail. It was proven that the ductility of the extruded alloys was improved by an increasing extrusion ratio, which is mainly attributed to by the weakened texture and the decreased dislocation density. In turn, Wang et al. [21] investigated the effects of different extrusion ratios on the AZ31 magnesium alloy bending products prepared by the staggered extrusion process.

A fine microstructure can generally be obtained by combining a low extrusion temperature with a high extrusion ratio R, which results in an improvement in the mechanical properties of the obtained alloys [22,23]. On the other hand, the increased extrusion temperature and the lowered extrusion ratio effectively reduce the amount of precipitate which has a positive effect on the improvement of ductility [24,25].

The extrusion ratio is also one of the key parameters in the production of composites [26,27,28]. The effects of extrusion ratio on microstructural evolution and mechanical behavior have been systematically investigated [29,30]. The research on MMC composites has showed that the micro-hardness and tensile strength of these composites increased with the growth of the extrusion ratio R. Moreover, it was shown that the wear resistance of elements from these hot-extruded composites was better for a higher extrusion ratio [31].

On the other hand, the studies [32] carried out on aluminum alloys have showed that when increasing the extrusion ratio R, both homogenized and non-homogenized AA-6063 alloys showed a growth in hardness and tensile strength, while elongation decreased. As the extrusion ratio R increases, the output speed of the profile from the extrusion press grows, provided that the speed of the main piston of the ram of this press is kept constant [33]. Statistical modeling of the hardness and roughness of the surface of cold-extruded aluminum in order to investigate the influence of the die angle, the extrusion ratio R, and lubrication on the hardness and roughness of the surface of the extruded products showed that the die angle had the greatest influence on the surface roughness of the manufactured products [34]. The extrusion ratio R also significantly influences the quality of bars extruded from hard-deformable aluminum alloys. In terms of the tested of extrusion ratio (R < 10), it was shown that extrusion with a higher R led to an increase in the dispersion of the strain intensity and temperature in the cross-section of the product, which in turn leads to greater differentiation in the material structure [32,35]. In addition, in the processes of obtaining profiles from recycling of metal chips, the extrusion ratio is a very important parameter. Studies have showed that in the case of extrusion of aluminum profiles from recycled aluminum chips, the strength and ductility of these profiles increased with the growth of the extrusion ratio [36].

The literature review shows that the publications presented so far in the field of the extrusion of metals and alloys mainly concern the research related to the first aspect concerning the influence of the extrusion ratio R on the operational, strength. and microstructure properties of the extruded products. The extrusion ratio R plays a key role in the industrial production of various materials, but its principle of influence is not very clear especially with regard to the load and wear of the tools. The noticeable lack of publications related to the second aspect concerning the influence of the extrusion ratio R on the strength and wear of the extrusion tools motivated us to undertake research in this field.

In the work on a specific example of concurrent extrusion, a qualitative comparative analysis was performed. Its purpose was to determine the relationship between the value of the extrusion ratio R and the load and wear intensity of the die. While maintaining a constant value of the outer diameter of the die (corresponding to the diameter of the charge), the distributions and values of equivalent stress, as well as deformations of the cross-sectional shape and displacement of measuring points on the die were compared depending on the extrusion ratio R. In addition, based on the obtained test results, the relationship between the extrusion ratio R and the maximum extrusion force was determined.

The subject of the influence of the extrusion ratio on the load and wear of the die in the extrusion process discussed in the article is important and current, both from the point of view of science and production practice. The extrusion ratio plays a key role in the industrial production of various materials, especially in relation to tool load and wear. The principle of its influence is not clear and there is insufficient information on this subject in the literature. The tests carried out in a wide range of the extrusion ratios have allowed for a qualitative analysis of its impact on the load and the wear of the die in the extrusion process to be conducted.

## 2. Experimental Research

**Figure 1:**
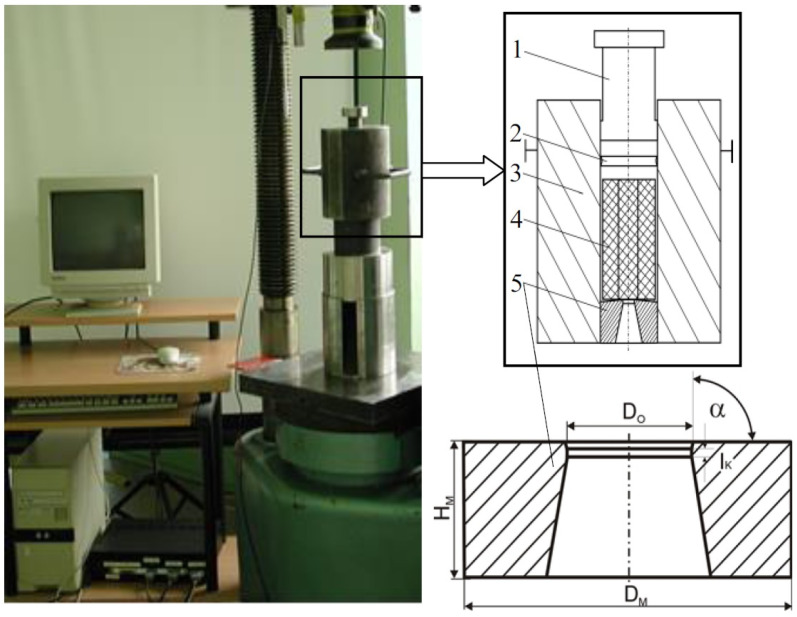
Experimental stand for the extrusion process and scheme of the die (1—punch, 2—pressure pad, 3—container, 4—billet, 5—die).

The experimental tests of the forward extrusion process were carried out on a hydraulic press using our own construction instrumentation. The tests were carried out using a flat single-hole die made of NC10 grade steel. The hardness of the surface of the die was 530 HV. The stand where the experimental extrusion tests were carried out was equipped with force sensors and displacements cooperating with the measuring transducer and a computer to acquire measurement data (force—punch displacement) is shown in Figure 1.

Table 1 summarizes the values of the basic extrusion parameters of the die used for testing, which are presented in Figure 1.

Hard lead was used as a research material. Some properties of the materials used in the research are presented in Table 2. The specimens used in the experimental research were cylinders of 32 mm diameter and 72 mm height. The extrusion process was conducted until about half of the billet length was extruded. Such an amount of extruded material allowed for a steady flow state to be achieved.

Based on the experimental data, the model was used to conduct a deeper theoretical analysis of the studied issue with a greater number of parameters, which allowed for determination of the impact of the extrusion ratio R on the intensity of the die wear and on the course and values of the extrusion force.

## 3. Numerical Analysis

**Figure 2:**
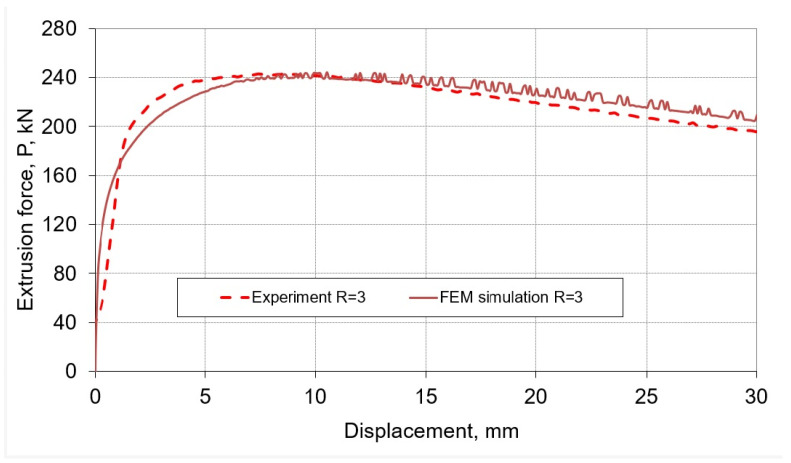
Experimental and numerical characteristics of the extrusion force—the displacement of the punch for the extrusion ratio R = 3.

As mentioned earlier, the test stand was equipped with a computer measuring system enabling determination of changes in the extrusion force depending on the displacement of the punch. On the basis of the experimental results obtained from the extrusion process, a load characteristics course was prepared, which was used to verify the correctness of the numerical model in the additional part of the study. An improvement of the convergence of the FEM model constructed in subsequent simulations was obtained by changing the calculation parameters, such as the size of the grid, the type and criteria of global remeshing, the size of the calculation step, etc. In order to make the results of the numerical calculations independent of the mesh size of bodies modeled as deformable, during the development of the FEM model, the influence of the finite element ship size was analyzed by densifying the mesh. After each compaction of the mesh, the extrusion force course was compared with the course of this force obtained with the use of the mesh before compaction. In this way, the nets were compacted until their further compaction had no greater impact on the compared parameter. A similar procedure was used to select the values of other parameters in the numerical model, which were sensitive to the modeling results. The results of the studies presented in the further part of the article were obtained using the FEM model, which showed the greatest convergence with the experiment (Figure 2).

**Figure 3:**
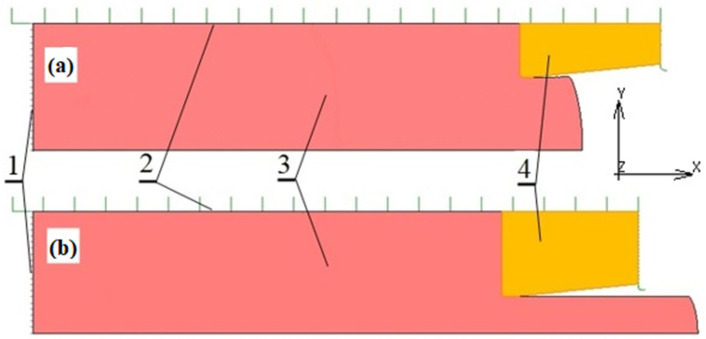
Axisymmetric geometrical model of the FEM simulation of extrusion process: (**a**) R = 3, (**b**) R = 11 (1—punch, 2—container, 3—billet, 4—die).

The numerical modeling of the concurrent extrusion process was performed using MSC MARC/Mentat software supporting the non-linear contact problems. The model built was an axially symmetrical two-dimensional one. Some examples of geometric models for FEM calculations with a 3.5 mm punch displacement for two different elongation ratios R = (3 and 11) are shown in Figure 3. It was assumed that the surfaces of punch one and container two were perfectly rigid, while extruded material three and die four were modeled as deformable bodies.

The mechanical properties of the die being deformed in the elastic range are as follows: E = 210,000 MPa and ν = 0.3. However, the properties of the extruded material were described using a rigid-plastic body model with non-linear strain hardening. The course of the strain hardening curve for the extruded material was described by the Hollomon equation in the form:

σ_pl_ = Kφ_i_^n^, (2)

The material constants for hard lead were K = 40 MPa, n = 0.23 [37]. The friction model was depicted using the Coulomb law. The friction coefficients were between the extruded material and tools and container μ = 0.25, between deformable die and container μ = 0.1. The tool hardness H in Equation (3) was the same as in the experiment. In order to create an FEM grid of extrusion material, class 4 type 10 elements were used—four-node, isoparametric, arbitrary quadrilateral elements written for axisymmetric applications [38]. Whereas to create an FEM grid of deformable die, class 3 type 2 elements were used—three-node, isoparametric, triangular elements. It is written for axisymmetric applications and uses bilinear interpolation functions [38]. The numerical simulation was performed using the global remeshing function. The evolution of the plasticity surface as a result of the strain hardening phenomenon was described using the isotropic model. The calculations used the Huber–Mises plasticity condition, and the associated Prandtl–Reuss plastic flow law. An implicit scheme for integrating differential equations by the Newton–Raphson method was used.

### 3.1. A Comparative Analysis of the Extrusion Force

**Figure 4:**
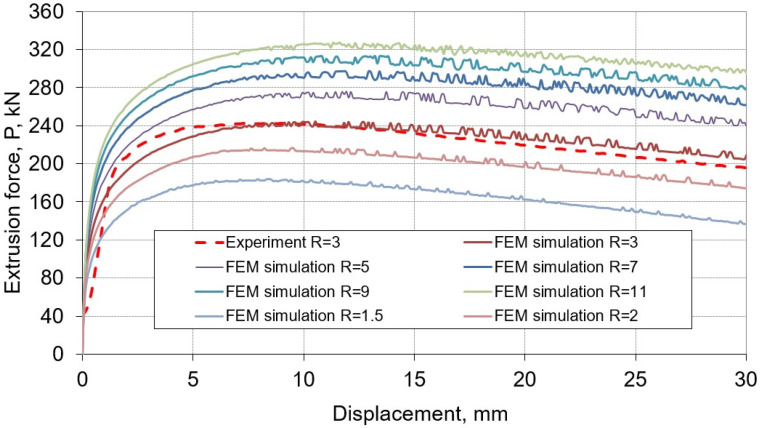
Experimental and numerical characteristics of the extrusion force—the displacement of the punch in the range of the tested values of the extrusion ratio R.

**Figure 5:**
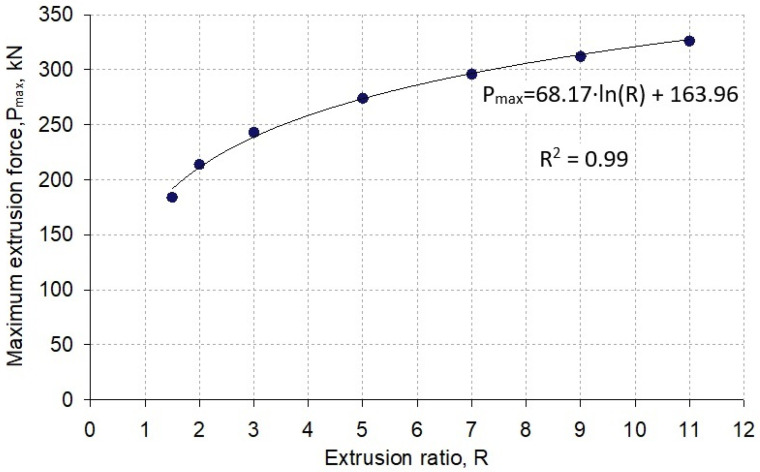
Dependence of the influence of the extrusion ratio R on the maximum extrusion force.

Numerical calculations were carried out for seven values of extrusion ratio, i.e., R = 1.5, R = 2, R = 3, R = 5, R = 7, R = 9, and R = 11. Figure 4 shows the courses between the displacement of the punch and the extrusion force obtained from numerical simulations and for the comparison with the curve obtained in experimental conditions. The shapes obtained from the calculation of the curves show the same character and are shifted relative to each other depending on the value of the extrusion ratio used. Studies have shown that there is a relationship between the extrusion ratio R and the maximum extrusion force. For the studied case, this relationship was presented on the graph (Figure 5) and approximated with a logarithmic equation.

While maintaining the same input material parameters, increasing the R extrusion ratio from 3 to 7 results in an approximate 1.2-fold increase in the maximum extrusion force. On the other hand, with an increasing R from 3 to 11, the calculated extrusion force increases more than 1.3 times. By analyzing the values of the extrusion ratio, i.e., increasing it by more than seven times in the entire examined range, it generates an increase in the maximum force value by almost two-fold. In the case of using an extrusion ratio in the range of 1.5 to 3, the difference in the maximum extrusion force is doubled compared to the use of larger extrusion ratio, i.e., 7 to 11.

### 3.2. Stresses and Die Deformation

**Figure 6:**
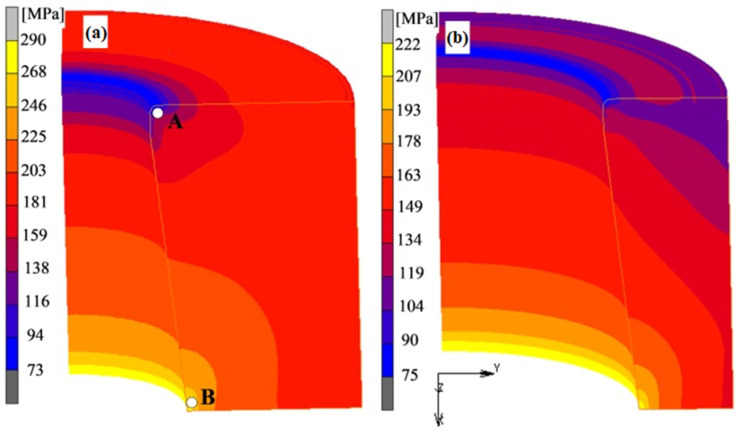
Distribution of Huber–Mises stress in the die during extrusion: (**a**) R = 11, (**b**) R = 3 (A and B—measuring points of stress in all models).

Figure 5 shows the distribution and values of Huber–Mises stresses in the die during extrusion with the ratio R = 11 and R = 3. The lowest values of equivalent stresses occur in the area of the die corner. In the case of R = 3 (Figure 6b), their average value is about 100 MPa. However, in the case of R = 11 (Figure 6a), their average value is about 110 MPa. It follows that the increase in the R ratio in the presented range slightly influences the magnitude of the equivalent stress in the area of the die corner. In turn, the stresses with the highest values occur in the area of the lower inner edge of the die, and their average values are about 222 MPa for R = 3 (Figure 6b) and about 290 MPa using R = 11 (Figure 6a).

**Figure 7:**
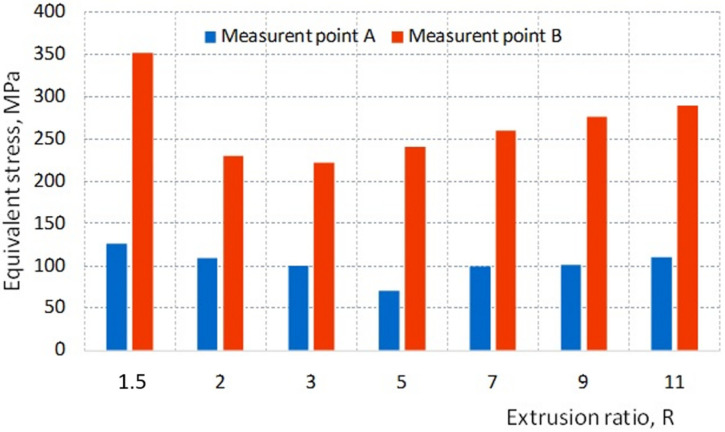
The impact of extrusion ratio R on the value of Huber–Mises stress at measuring points A and B on the longitudinal section of the die.

In order to compare the stress values over the entire range of the extrusion ratio values tested, the stress analysis was performed by selecting measuring points A and B in these two characteristic areas of the die (Figure 6a). The stress values calculated at selected measuring points depending on the R ratio are presented on the graph (Figure 7).

**Figure 8:**
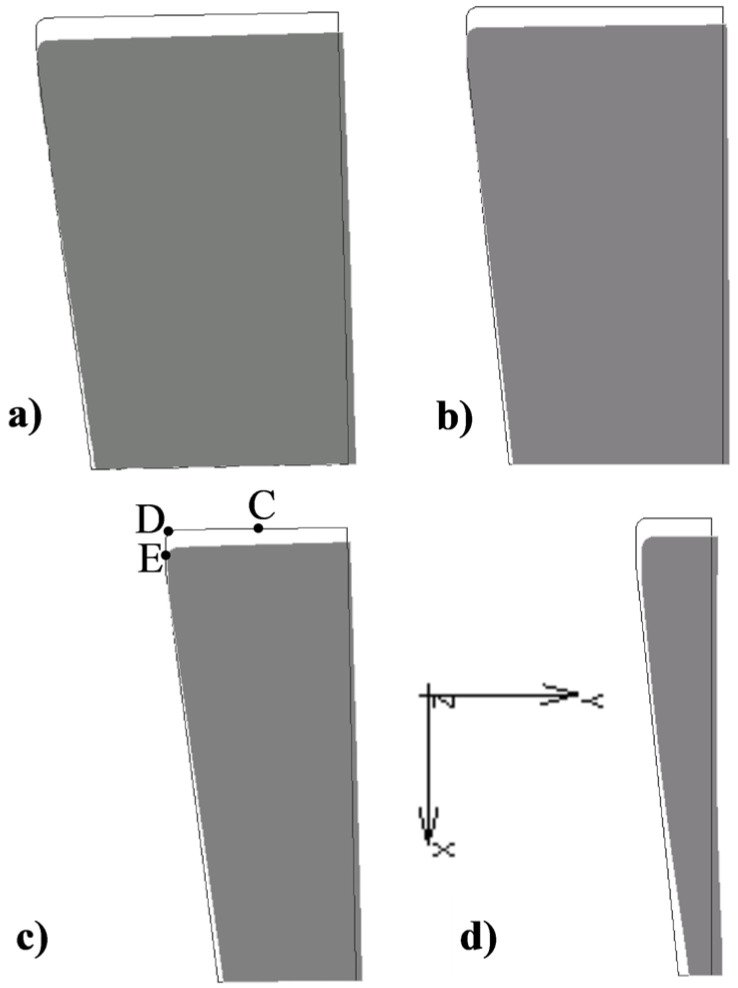
Die deformation during extrusion: (**a**) R = 11, (**b**) R = 7, (**c**) R = 3, (**d**) R = 1.5 [(def. factor ×50) C, D and E—measuring points of displacement Ux and Uy in all models].

The stresses at point A (blue columns in Figure 7) change with the R ratio in a relatively small range. At the same time, an increase in the R ratio in the range from 1.5 to 5 results in a decrease in their value from 126 to 74 MPa. This means that in spite of the increase in extrusion force together with the R ratio in this range, an increase in the cross-section of the die with excess compensates for the effort of the material, which is the reason for a slight decrease in the value of equivalent stresses. In turn, increasing the R ratio further in the range of 5 to 11 causes a slight increase in stress at point A. This means that the increase in the cross-section of the die in this range does not fully compensate for the increase in the load on the die during extrusion. A similar tendency to change the stress is observed at point B. However, at this point, the lowest stress value corresponds to the ratio R = 3 and is 222 MPa. In the R range from 3 to 11, the stress increases almost proportionally reaching the value of 290 MPa for R = 11. As you can see in the graph (Figure 7), the value of stress calculated at point B for the ratio R = 1.5 is much higher than the others and amounts to 352 MPa. Such a high stress value (in spite of the least extrusion force) is caused by too low a die cross-section that does not provide sufficient rigidity (Figure 8d) compared to other tested cross-sections (Figure 8a–c).

Due to the different die deformations depending on the R ratio, displacement of the selected die points (Figure 8c) in the axial Ux (points D and C) and radial Uy (points D and E) were analyzed.

**Figure 9:**
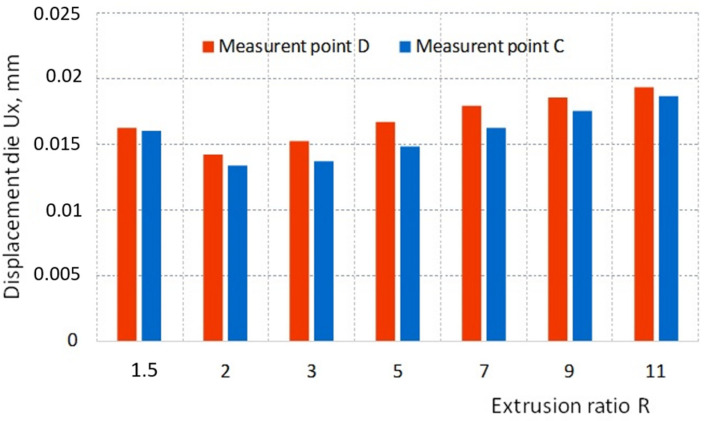
The impact of the extrusion ratio R on the axial displacement Ux of the die at points C and D.

The value of the displacement in the axial direction of the die, regardless of the R ratio, is slightly higher at point D than at point C (Figure 9). The smallest axial displacement at points D and C occurs when R = 2 and increases almost proportionally reaching 0.018 mm for R = 11.

**Figure 10:**
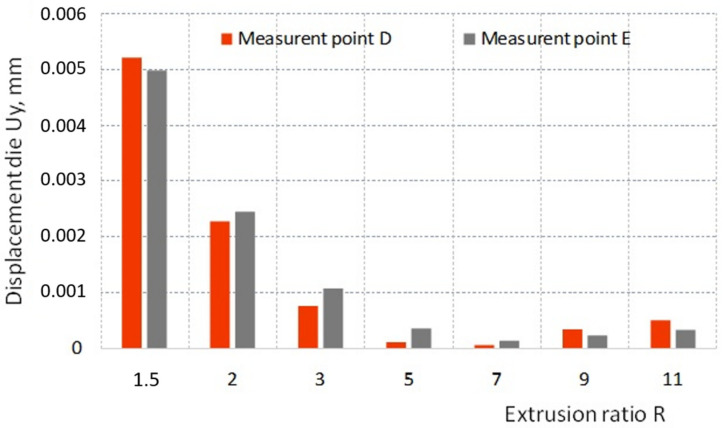
The impact of extrusion ratio R on the radial displacement Uy of the die at points D and E.

From a practical point of view, it is much more important to displace the corner and the calibration strip of the die in the radial direction because it directly affects the geometric parameters of the extruded product. For the case analyzed, the displacement of measuring points D and E under the influence of load in the radial direction of the die depending on the R ratio is shown in the graph (Figure 10).

By far the highest values of these displacements occur for the die at R = 1.5, which, as already mentioned, results from too small cross-section, which does not guarantee sufficient rigidity of the tool. However, a slight increase in the ratio to R = 2 caused an about two-fold reduction in axial displacement at measuring points D and E. The lowest values of radial displacement at the points analyzed were noted for the ratio R = 7. Slightly less favorable in this ranking were the ratios R = 5, 9, and 11.

### 3.3. A Comparative Analysis of Tool Wear

To calculate the wear value, the Archard model [39] was used, according to which:

(3) $$dV=k\frac{dFdL}{H},$$

where:

*dV*—volume wear
*k*—wear coefficient
*dF*—normal force
*dL*—sliding distance
*H*—hardness

In the research performed, the die wear profile during the extrusion was calculated by implementing the Archard FEM-based wear model in MARC 2020 software as presented below:

(4) $$\dot{w}=\frac{k}{H}\sigma_{n}v_{rel},$$

where:

$\dot{w}$—rate of change of wear in the direction normal to the surface
*σ_n_*—normal stress in contact
*v_rel_*—relative sliding velocity

The wear amount indicated as the wear depth was calculated using the equation as follows:

(5) $$w_{n+1}=w_{n}+\dot{w}\Delta t,$$

where:

*w_n_*_+1_—current wear depth
*w_n_*—wear depth amount in the previous computation step
$\dot{w}\Delta t$—incremental wear
Δt—time in the computation step

The tool wear indicator can be used as a criterion to assess tool life, and in the analyzed case for comparative analysis of the impact of the extrusion factor on the amount of tool wear. In the tests conducted, the value of the wear factor k was assumed to be 10^−4^ [40].

**Figure 11:**
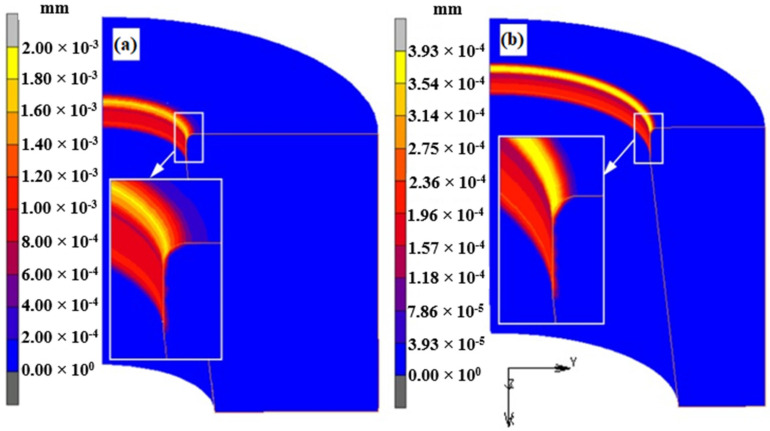
Die areas with the highest wear intensity (wear depth distribution): (**a**) R = 11, (**b**) R = 3.

The comparative analysis of the wear was carried out in the area of the corner of the die and the calibration strip because the wear is the highest there (Figure 11). The distributions and values of the wear depth presented show that the largest wear depth occurs in the middle part of the die corner radius and for extrusion with the ratio R = 11 (Figure 11a) is almost 4.5 times greater than for R = 3 (Figure 11b).

**Figure 12:**
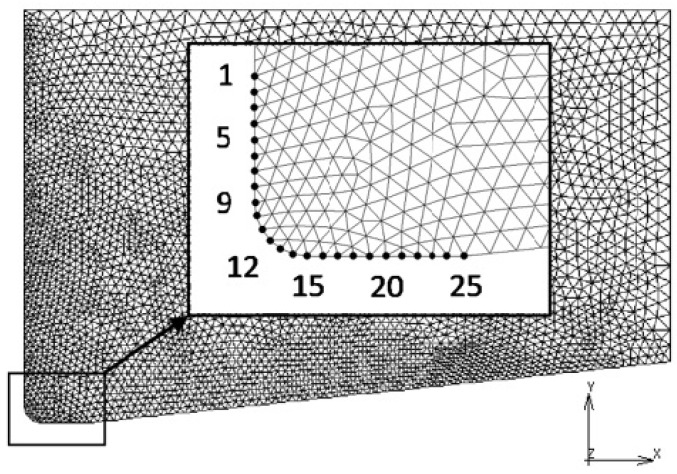
Region and measuring points of wear depth in all models.

In order to conduct a comparative analysis of the impact of the R ratio on the intensity of the die wear in the range examined, the amount of the wear was analyzed at 25 measuring points lying in the corner and the die calibration strip (Figure 12).

**Figure 13:**
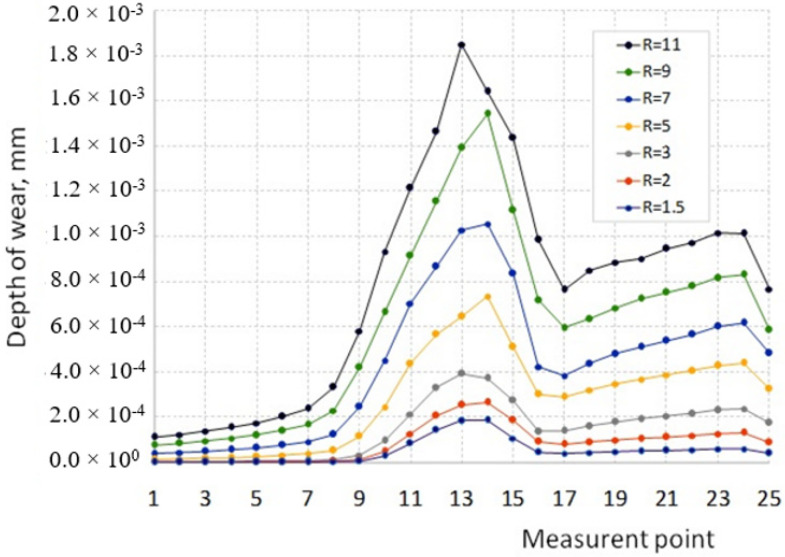
The impact of the extrusion ratio R on the depth of die wear in the measuring area.

The depth of wear at individual points along the measurement length within the range of the tested R-values is presented in the graph (Figure 13).

Within all tested values of the R ratio, the smallest wear occurs in the area of the corner on the face of the die, and its depth increases in the direction of the radius of the die corner (points 1 to 8). However, the largest wear depth is in the corner of the die (points 9 to 15), and the maximum wear depth lies in the center of this radius (points 13 and 14). The depth of the wear in the area of the calibration strip (points 16 to 25) is definitely greater than on the front face but is correspondingly smaller than on the die radius. In all measuring areas (points from 1 to 25), the wear depth increases as the R value increases. At the same time, the increase in wear depth is not proportional to the value of the R ratio but increases more with the higher the value of this ratio. In other words, in the R ratio range from 1.5 to 3, the increase in wear depth is not as intense as, e.g., in the R ratio range from 3 to 11. For example, while maintaining the same input material parameters, increasing the R ratio from 1.5 to 3 results in a more than two-fold increase in the maximum wear depth. However, increasing the R ratio from 3 to 7 causes a more than 2.5-fold increase in the depth of tool wear. On the other hand, when the R increases from 3 to 11, the maximum wear depth grows more than 4.5 times. As can be seen in the results from the data on the graph (Figure 12) for small values of the R ratio along the length of the calibration strip (points 17 to 24), the variation in the depth of wear is small. However, as the R-ratio increases, this difference along the length of the calibration strip increases, i.e., the difference in wear depth between points 17 and 24 increases. This is most likely due to the change in the nature of the metal flow depending on the value of the R ratio.

## 4. Conclusions

The extrusion ratio R is the basic parameter of any extrusion process. In this paper, with regard to the example of concurrent extrusion of a round bar, the results of a qualitative FEM analysis of the impact of the extrusion ratio R on process parameters such as die wear intensity, extrusion force, stress intensity in the die, and its elastic deformation during extrusion are presented. The tests were carried out in a wide range of the R (1.5–11), assuming a constant outer diameter of the die. A comparative analysis of wear was carried out in the area of the die corner radius and the calibration strip since the wear is the highest there. Based on the research and the analysis, the following conclusions can be drawn:

(1) As the value of the extrusion ratio R increases, the extrusion force grows, while the increase in this force is not proportional to the increase in the R ratio. The most intensive increase in force was observed in the range of small ratios R ≤ 3. Along with a further increase in the value of the R ratio, the intensity of the extrusion force became smaller. It has been shown that the relationship between the extrusion ratio R and the maximum extrusion force P_max_ can be described by a logarithmic equation in the form: P_max_ = A∙ln(R) + B, in which A and B are constants that can be determined experimentally or on the basis of numerical simulations of the extrusion process.
(2) Assuming a constant outer diameter of the die corresponding to the diameter of the extrusion charge, the most advantageous value due to its deformation is the use of an extrusion ratio R greater than or equal to 3. In the case of smaller values of R ratios, the die cross-section may not provide adequate rigidity. In addition, it was shown that the value of equivalent stresses in the area of the die corner depends relatively little on the value of the extrusion ratio R.
(3) The use of the Archard wear model in the numerical simulation made it possible to conduct a comparative analysis of the impact of the extrusion ratio R on the intensity of the die wear. The die wear was the greatest in the corner radius. The average depth of die wear in the analyzed area largely depends on the R extrusion ratio. An increase in the R ratio value is accompanied by an increase in the die load and an increase in the flow speed extruded through the metal die, which causes an intensive increase in its wear. Based on the results obtained, it can be estimated that, e.g., increasing the R ratio from 3 to 7 causes an over 2.5-fold increase in tool wear. In turn, the implementation of the extrusion process using R = 11 results in an up to 4.5-fold increase in the average depth of die wear compared to its consumption when the extrusion ratio R = 3.

## Figures and Tables

**Table 1:** Basing dimensions of dies used in the extrusion process.

Parameter	Value
Die reduction angle α, degrees	90
Die external diameter D_M,_ mm	36
Die height H_M_, mm	20
Extrusion ratio, R	1.5, 2, 3, 5, 7, 9, 11
Length of the calibrating part l_k_, mm	2
Rounding radius in the calibrating part r, mm	0.5

**Table 2:** Some features of materials used in the investigations.

Material	Chemical Composition %	Yield Stress MPa	Brinell Hardness HB
Hard lead	(2.5–5.0) Sb; 0.015 As; 0.04 Cu; 0.0012 Fe; 0.01 Bi	10	8.7
